# Extranodal Intra-abdominal Histiocytic Sarcoma Initially Diagnosed as Abdominal Tuberculosis

**DOI:** 10.7759/cureus.112203

**Published:** 2026-07-07

**Authors:** Guadalupe de los Angeles Salazar Gonzalez, Valeria Gonzalez Quiroz, Cesar Fabian Vallejo Rico, Jorge Alberto Garay Hernandez, Alicia Marali Ibarra Moncivais

**Affiliations:** 1 Internal Medicine, Instituto Mexicano del Seguro Social, Durango, MEX

**Keywords:** differencial diagnosis, extranodal neoplasm, histiocytic sarcoma, immunohistochemistry(ihc), peritoneal tuberculosis

## Abstract

Histiocytic sarcoma (HS) is a rare and aggressive hematolymphoid neoplasm that frequently presents as extranodal disease. Intra-abdominal involvement is exceedingly rare and may clinically resemble abdominal tuberculosis. We present the case of a 29-year-old woman with abdominal pain, unintentional weight loss of 15 kg (25% of her baseline body weight) over four months, and altered bowel habits. CT demonstrated a large intra-abdominal mass measuring 14 × 11 × 7 cm with internal calcifications and mass effect on adjacent structures. Initial diagnostic evaluation included image-guided fine-needle aspiration followed by diagnostic laparoscopic biopsy, which demonstrated granulomatous inflammation with Ziehl-Neelsen positivity.

Given the patient's constitutional symptoms, abdominal imaging findings, granulomatous inflammation with Ziehl-Neelsen positivity on the initial biopsy, and a positive interferon-gamma release assay (IGRA), a presumptive diagnosis of abdominal tuberculosis was established by the multidisciplinary team, and empirical antituberculous therapy was initiated while further diagnostic evaluation continued. Despite treatment, the patient experienced clinical deterioration and radiological progression. Exploratory laparotomy followed by histopathological and immunohistochemical evaluation confirmed extranodal HS. This report illustrates the diagnostic challenge posed by this rare malignancy, particularly when it presents with constitutional symptoms, nonspecific radiological findings, and initial histopathological features suggestive of abdominal tuberculosis. It emphasizes the importance of early tissue diagnosis through comprehensive histopathological evaluation to avoid delays in appropriate oncologic management.

## Introduction

Histiocytic sarcoma (HS) is an exceptionally rare malignant hematopoietic neoplasm derived from mature histiocytes. Due to its extremely low incidence, estimated at fewer than one case per million inhabitants, HS is considered one of the rarest hematolymphoid malignancies [1,2]. The fifth edition of the World Health Organization (WHO) Classification of Hematolymphoid Tumours recognizes HS within the spectrum of histiocytic and dendritic cell neoplasms and defines it by morphologic and immunophenotypic evidence of histiocytic differentiation [3].

Owing to its rarity, the biological behavior and optimal management of HS remain incompletely understood [4]. However, available evidence indicates an aggressive clinical course with frequent extranodal involvement and a poor overall prognosis, particularly in disseminated disease [5]. Extranodal manifestations are common and may involve the gastrointestinal tract, spleen, liver, skin, soft tissues, and central nervous system [6]. The differential diagnosis of intra-abdominal masses includes both neoplastic and infectious diseases, particularly tuberculosis, which may closely mimic malignant processes on clinical and radiological evaluation [7,8].

The diagnosis is often challenging because clinical manifestations and imaging findings are frequently nonspecific, requiring correlation with microbiological and histopathological data. Furthermore, extrapulmonary tuberculosis may mimic a wide range of infectious, inflammatory, and malignant conditions, including lymphoma, carcinomatosis, and granulomatous diseases [9]. Histopathological examination with immunohistochemical studies remains essential for the diagnosis of histiocytic neoplasms. Cells of the histiocytic/macrophage lineage typically express markers such as CD68, CD163, and CD45, which help establish their histiocytic origin [6]. Intra-abdominal tuberculosis is considered an important differential diagnosis, as it can present with constitutional symptoms, weight loss, lymphadenopathy, ascites, and peritoneal masses, closely mimicking intra-abdominal neoplasms. This overlap can lead to considerable diagnostic uncertainty, especially in tuberculosis-endemic regions [10].

Furthermore, immunological tests such as interferon-gamma release assays (IGRAs) cannot reliably distinguish latent tuberculosis infection from active disease and therefore should not be used as a substitute for tissue diagnosis. In cases with atypical clinical or radiological features, histopathological confirmation remains essential for establishing an accurate diagnosis [11]. Currently, no standard treatment has been established for HS because of its rarity. Management is individualized according to disease extent and may include complete surgical resection for localized disease, systemic chemotherapy, radiotherapy, and targeted therapy in selected patients with actionable molecular alterations. Despite treatment, disseminated disease is associated with an aggressive clinical course and poor prognosis [4,5].

We report a case of extranodal HS presenting as a large intra-abdominal mass that was initially managed as presumed abdominal tuberculosis based on the patient’s constitutional symptoms, imaging findings, granulomatous inflammation with Ziehl-Neelsen positivity on the initial biopsy, and a positive IGRA result. The diagnosis was ultimately established by surgical biopsy and immunohistochemical analysis.

## Case presentation

A 29-year-old woman with a history of hypothyroidism managed with levothyroxine replacement therapy presented with a three-month history of diffuse abdominal pain, altered bowel habits, and unintentional weight loss of 15 kg (25% of her baseline body weight) over the preceding four months. On physical examination, the abdomen was soft and non-distended, with a palpable mesogastric mass associated with deep tenderness on palpation. No signs of peritoneal irritation or palpable peripheral lymphadenopathy were identified. Laboratory evaluation demonstrated mild leukocytosis and elevated inflammatory markers, while liver and renal function tests were within normal limits (Table 1).

**Table 1:** Laboratory studies

Variables	Results	Units	Normal parameters
Leukocytes	11.5	x 10⁹/L	(4-11)
Erythrocytes	4.41	x 10^12^/L	(4-5)
Hemoglobin	12.1	g/dL	(12-17)
Hematocrit	36.7	%	(35-52)
Mean corpuscular volume	83.2	fL	(80-100)
Mean corpuscular hemoglobin	27.4	pg/cell	(27-31.2)
Platelets	212	× 10³/mm³	(130-400)
Neutrophils #	8.77	× 10³/mm³	(2-8)
Lymphocytes #	0.93	× 10³/mm³	(1-5)
Eosinophils #	0.16	× 10³/mm³	(0-0.40)
Basophils #	0.05	× 10³/mm³	(0-0.20)
Glucose	96	mg/dl	(74-106)
Urea	15	mg/dl	(19-43)
Urea nitrogen	7	mg/dl	(9.0-20)
Creatinine	0.5	mg/dl	(0.60-1.20)
Total proteins	7.1	g/dL	(6.3-8.2)
Albumin	4.2	g/dL	(3.5-5.0)
Total bilirubin	0.6	mg/dl	(0.2-1.30)
Indirect bilirubin	0.4	mg/dl	(0-1.10)
Direct bilirubin	0	mg/dl	(0-1)
Aspartate aminotransferase	17	U/L	(14-36)
Alanine aminotransferase	20	U/L	(< 35)
Alkaline phosphatase	171	U/L	(38-126)
Lactate dehydrogenase	127	U/L	(120-246)
Sodium	137	mmol/l	(137-145)
Potassium	4.2	mmol/l	(3.5-5.1)
Serum chloride	102	mmol/l	(98-107)
Calcium	9.7	mg/dl	(8.4-10.2)
Phosphorus	4.1	mg/dl	(2.5-4.5)
Magnesium	2	mg/dl	(1.6-2.4)
Prothrombin time	16	Seconds	(11.9-15.6)
Prothrombin time witness	13.5	Seconds	
International standardized index	1.21		
Partial thromboplastin time	38.2	Seconds	(32.22-40.14)
Partial thromboplastin time index	36.18	Seconds	

Abdominal ultrasonography revealed a large intra-abdominal mass. Non-contrast CT of the abdomen demonstrated a large heterogeneous intra-abdominal mass measuring 14 × 11 × 7 cm with internal calcifications and mass effect on adjacent structures, without radiologically significant lymphadenopathy (Figure 1). Colonoscopy was performed to exclude a primary colorectal neoplasm and assess possible colonic invasion because of the close anatomical relationship between the mass and the colon on imaging. It demonstrated extrinsic compression without evidence of mucosal involvement, supporting an extraintestinal origin of the lesion.

**Figure 1:**
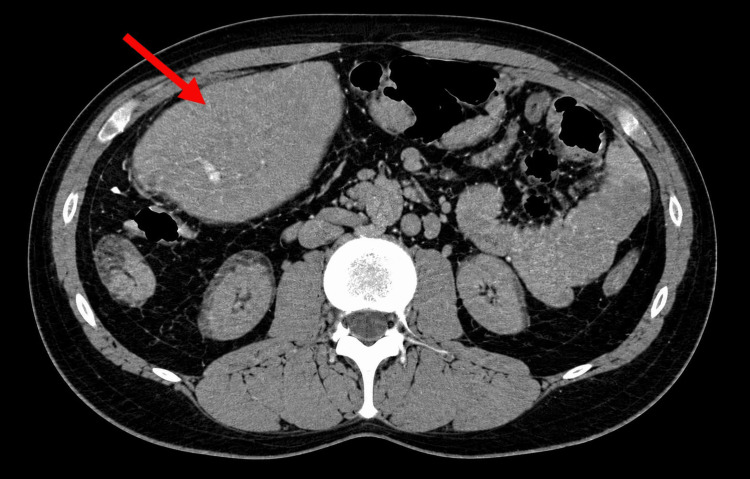
Non-contrast abdominal CT demonstrating a large heterogeneous intra-abdominal mass with internal calcifications Axial non-contrast CT image of the abdomen showing a large heterogeneous intra-abdominal soft-tissue mass with internal calcifications, associated with mass effect on adjacent structures. These findings initially raised suspicion for an infectious or neoplastic intra-abdominal process and prompted further diagnostic evaluation CT: computed tomography

Given the imaging findings, the patient underwent an image-guided fine-needle aspiration biopsy. Histopathological examination demonstrated histiocytic proliferation associated with chronic granulomatous inflammation, giant cells, and Ziehl-Neelsen-positive acid-fast bacilli, suggesting mycobacterial infection. A subsequent diagnostic laparoscopic biopsy yielded similar findings. Considering these histopathological findings together with the patient's constitutional symptoms, abdominal imaging findings, and a positive IGRA, a multidisciplinary team established a presumptive diagnosis of abdominal tuberculosis, and empirical antituberculous therapy was initiated while further diagnostic evaluation continued.

Despite treatment, the patient experienced persistent symptoms and progressive clinical deterioration. Follow-up imaging demonstrated interval enlargement of the intra-abdominal mass without radiological response to empirical antituberculous therapy (Figure 2). This lack of clinical and radiological improvement prompted repeat tissue sampling by exploratory laparotomy.

**Figure 2:**
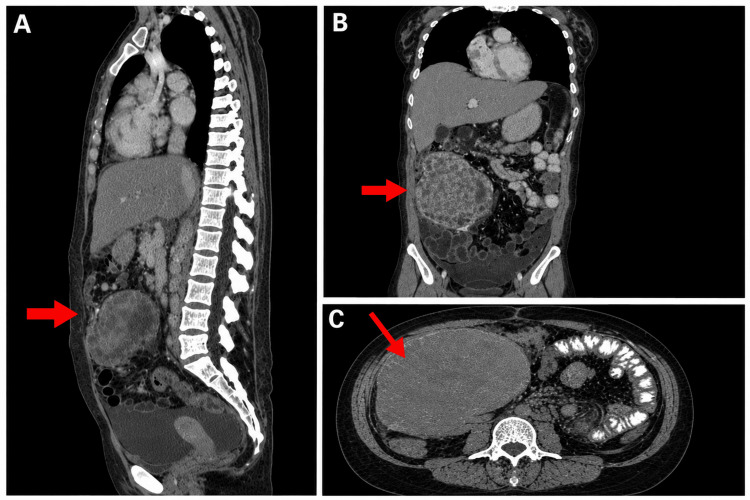
Follow-up non-contrast CT demonstrating progression of the intra-abdominal mass (A) Sagittal, (B) coronal, and (C) axial CT images demonstrating interval enlargement of the heterogeneous intra-abdominal mass with increased mass effect on adjacent structures, indicating radiological disease progression despite empirical antituberculous therapy. The absence of clinical and radiological improvement prompted repeat tissue diagnosis by exploratory laparotomy CT: computed tomography

Intraoperative findings revealed a large infiltrative intra-abdominal mass densely adherent to adjacent structures. Because of its extensive local involvement, complete surgical excision was not feasible; therefore, representative tissue samples were obtained during exploratory laparotomy for definitive histopathological diagnosis (Figure 3).

**Figure 3:**
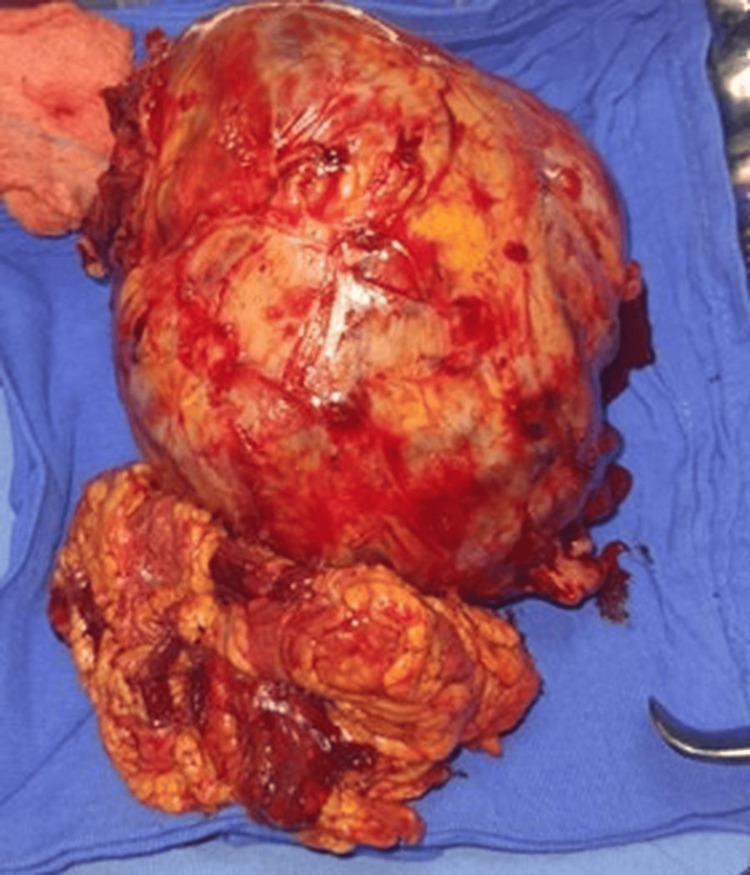
Gross surgical specimen of a large multinodular abdominal mass Gross pathological specimen obtained during exploratory laparotomy showing a large multinodular intra-abdominal mass measuring 14 × 11 × 7 cm with a smooth external surface and areas of hemorrhagic congestion. Adherent adipose tissue is present. Histopathological and immunohistochemical evaluation subsequently confirmed extranodal histiocytic sarcoma

Histopathological examination revealed a spindle-cell neoplasm arranged in intersecting fascicles and storiform areas. The tumor cells exhibited eosinophilic cytoplasm and elongated to oval nuclei with variable atypia. Immunohistochemical analysis showed diffuse positivity for CD163 and CD68, focal positivity for CD45 and CD21, and negative staining for CD20, CD1a, and S100. Together with the characteristic histomorphological features, these findings supported histiocytic differentiation and the diagnosis of extranodal HS (Figures 4, 5).

**Figure 4:**
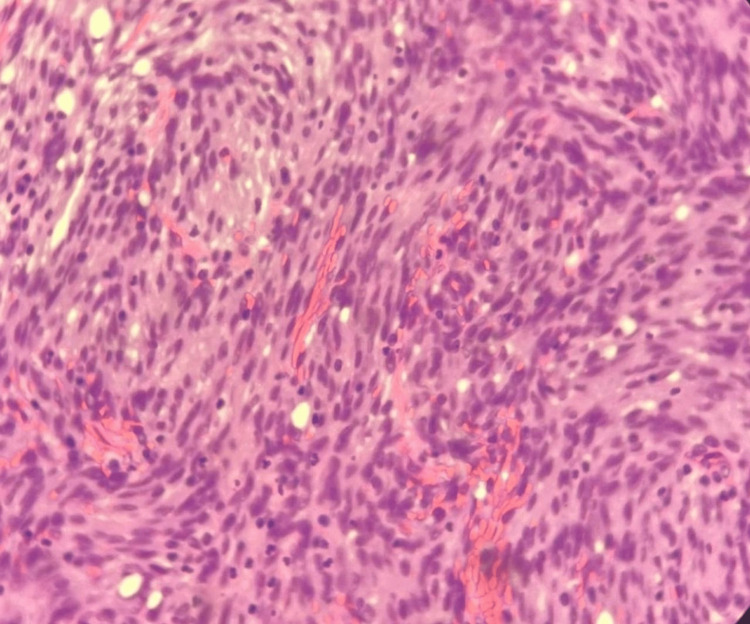
Histopathological features of histiocytic sarcoma Hematoxylin and eosin staining demonstrating a diffuse proliferation of pleomorphic neoplastic cells arranged in short fascicles, with abundant eosinophilic cytoplasm and irregular hyperchromatic nuclei (original magnification x400)

**Figure 5:**
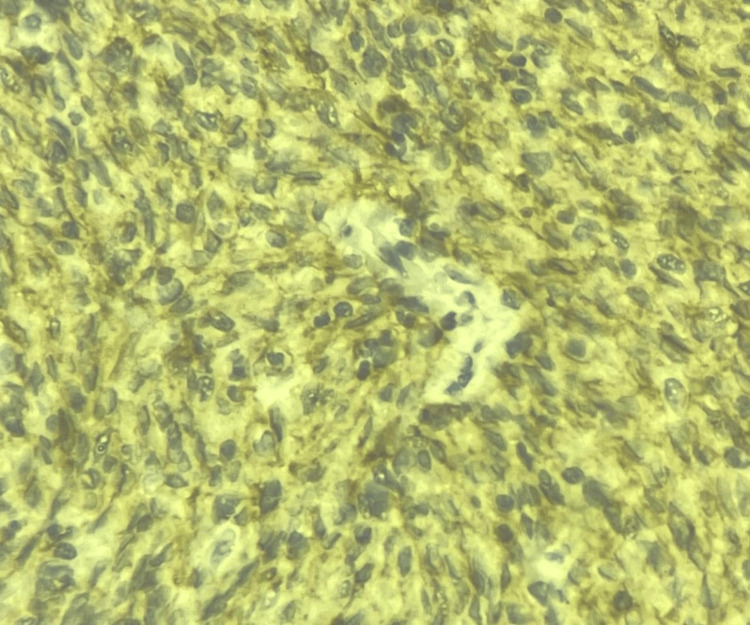
CD163 expression in tumor cells Immunohistochemical staining showing diffuse cytoplasmic positivity for CD163 in the neoplastic cells, supporting histiocytic differentiation (original magnification 400x)

Complete surgical excision was not feasible because the tumor was considered unresectable at exploratory laparotomy; therefore, surgical margins could not be assessed. PET/CT was not performed during the staging work-up. Following multidisciplinary oncologic evaluation, the patient was considered to have advanced unresectable disease and received first-line systemic treatment with liposomal doxorubicin. Five cycles were administered; however, no clinical or radiological response was observed. Treatment response was assessed by serial clinical evaluation and follow-up non-contrast CT, which demonstrated disease progression. Radiotherapy was subsequently initiated after progression. At the time of manuscript preparation, the patient had completed six months of oncologic follow-up.

## Discussion

This case report illustrates the diagnostic difficulty of extranodal HS when presenting as an intra-abdominal mass [1,2]. In regions where tuberculosis remains endemic, constitutional symptoms and nonspecific imaging findings may initially suggest abdominal tuberculosis, particularly when accompanied by supportive microbiological or immunological findings [12]. However, definitive diagnosis requires histopathological examination with appropriate immunohistochemical evaluation [1,2].

IGRAs should be interpreted with caution. Although they provide evidence of immune sensitization to Mycobacterium tuberculosis, they cannot differentiate latent tuberculosis infection from active disease. Consequently, positive IGRA results should not replace microbiological or histopathological confirmation, particularly when imaging findings are atypical or when disease progression occurs despite empirical antituberculous therapy [13,14]. In the present case, lack of clinical and radiological response to empirical antituberculous therapy prompted repeat tissue sampling through exploratory laparotomy, ultimately establishing the diagnosis. In retrospect, comprehensive staging and repeat tissue diagnosis before prolonged empirical therapy might have facilitated earlier oncologic management. Complete surgical resection was not feasible as the tumor was considered unresectable at surgical exploration. These limitations should be interpreted in the context of real-world clinical practice and are acknowledged as limitations of this report.

HS most commonly presents as extranodal disease. Bellalah et al. reported an aggressive case involving the small bowel and lung that presented as acute peritonitis secondary to intestinal perforation, highlighting that abdominal HS may mimic more common surgical emergencies and that definitive diagnosis often requires histopathological evaluation [15]. The present case adds to the limited literature on abdominal HS by documenting an intra-abdominal presentation initially treated as tuberculosis. Recent reports continue to emphasize the rarity of abdominal HS, with the available evidence consisting predominantly of isolated case reports involving gastrointestinal or multiple abdominal sites. The scarcity of reported intra-abdominal presentations highlights the diagnostic complexity of this uncommon manifestation [7]. Its relevance lies not only in the rarity of the tumor location but also in the diagnostic pitfall created by a positive IGRA in the context of compatible constitutional symptoms.

Definitive diagnosis of HS relies on histopathological examination together with immunohistochemical confirmation of histiocytic differentiation. Histologically, the tumor is composed of large atypical pleomorphic cells with abundant eosinophilic cytoplasm arranged in intersecting fascicles and storiform areas. Because HS shows considerable morphologic overlap with lymphomas, carcinomas, melanomas, dendritic cell neoplasms, and pleomorphic sarcomas, immunohistochemistry is essential for establishing the diagnosis and excluding its major mimickers [16]. The diagnosis is based on the integration of morphologic features and immunophenotypic findings rather than on any single immunohistochemical marker. Among histiocytic markers, CD163 is currently considered the most specific marker for histiocytic differentiation, whereas CD68 is highly sensitive but less specific [17]. In the present case, diffuse expression of CD163 and CD68, focal expression of CD45 and CD21, together with negative staining for CD20, CD1a, and S100, supported the diagnosis of extranodal HS in the appropriate histopathological context. 

One limitation of this case report is that an extended immunohistochemical panel was not available at our institution. Additional markers to exclude epithelial, mesenchymal, neural, melanocytic, vascular, and dendritic cell neoplasms could not be performed. Nevertheless, the diagnosis was established based on the characteristic histopathological findings together with the available immunophenotypic profile, as interpreted by experienced pathologists. Another limitation is that complete oncologic staging could not be performed because PET/CT was not available at our institution. Consequently, metabolic staging before definitive diagnosis was not possible, and disease extent was assessed using conventional imaging and clinical evaluation.

Because HS is an exceptionally rare malignancy, there is no universally accepted standard treatment strategy, and current recommendations are largely based on retrospective studies, case reports, and expert consensus. According to the National Comprehensive Cancer Network (NCCN) Guidelines for Histiocytic Neoplasms, treatment should be individualized according to disease extent, organ involvement, clinical presentation, and the presence of actionable molecular alterations. Localized disease may be managed with surgery and/or radiotherapy, whereas multifocal or disseminated disease generally requires systemic therapy, including consideration of targeted therapies in selected patients [18].

Molecular profiling was not available at our institution; therefore, actionable genomic alterations could not be evaluated in this patient. Nevertheless, comprehensive genomic testing should be considered whenever feasible, as alterations involving the MAPK pathway, including BRAF, MAP2K1, KRAS, and NRAS, may identify candidates for targeted therapies. For patients with advanced unresectable disease, first-line systemic therapy is therefore individualized according to disease extent, resectability, patient characteristics, and institutional experience.

Disseminated HS is associated with an unfavorable prognosis, reflecting the aggressive biological behavior of the disease and the limited effectiveness of available systemic therapies. Reported median overall survival remains approximately six months despite treatment [17]. Our patient had advanced unresectable intra-abdominal disease and showed progression despite five cycles of liposomal doxorubicin. This clinical course is consistent with the aggressive behavior reported in extranodal HS and highlights the importance of early tissue diagnosis, prompt staging, and consideration of molecular testing. In retrospect, image-guided biopsy followed by complete staging before surgical exploration might have represented a more appropriate diagnostic strategy in this patient. Exploratory laparotomy was undertaken because of persistent clinical deterioration and radiological progression despite empirical antituberculous therapy, with the primary objective of obtaining adequate tissue for definitive diagnosis rather than therapeutic resection. Because complete surgical excision was not feasible, adjuvant radiotherapy in the conventional postoperative setting was not applicable. Initial management therefore consisted of systemic therapy, whereas radiotherapy was introduced only after documented disease progression.

This case report emphasizes three important clinical lessons. First, extranodal HS presenting as an intra-abdominal mass may clinically resemble abdominal tuberculosis when constitutional symptoms, nonspecific imaging findings, and supportive microbiological or immunological results coexist. Second, a positive IGRA should not be considered diagnostic of active tuberculosis without compatible microbiological or histopathological evidence. Third, persistent clinical or radiological progression despite empirical therapy should prompt early repeat tissue diagnosis and multidisciplinary reassessment. Earlier histopathological confirmation may reduce diagnostic delay and facilitate timely oncologic management.

Literature review and comparison with previously reported cases

Because extranodal HS involving intra-abdominal soft tissues is exceedingly uncommon, we reviewed previously published cases to compare their clinical presentation, diagnostic approach, treatment strategies, and outcomes. Similar to previously reported patients, our case presented with nonspecific constitutional and abdominal symptoms requiring histopathological and immunohistochemical confirmation. A comparison of representative cases from the literature is summarized in Table 2 [15,19,20].

**Table 2:** Comparison of previously reported cases* of intra-abdominal HS with the present case ^*^[15,19,20] HS: histiocytic sarcoma; IGRA: interferon-gamma release assay

Country	Year	Sex	Age in years	Brief description
Tunisia	2021	Female	37	HS presenting as an acute abdominal condition mimicking peritonitis
United States	2005	Female	49	Mesenteric mass associated with constitutional symptoms, with delayed diagnosis
Italy	2010	Male	58	Gastrointestinal HS with secondary peritoneal involvement
Present case	2026	Female	29	Extranodal intra-abdominal HS initially managed as presumed abdominal tuberculosis because of constitutional symptoms, granulomatous inflammation with Ziehl-Neelsen positivity on the initial biopsy, and a positive IGRA, with subsequent disease progression despite antituberculous therapy

## Conclusions

Extranodal HS presenting as an intra-abdominal mass is an exceptionally rare and aggressive malignancy. In this case, the initial presumptive diagnosis of abdominal tuberculosis was influenced by the combination of constitutional symptoms, a positive IGRA, and initial histopathological findings of granulomatous inflammation with Ziehl-Neelsen positivity rather than by radiological features typical of peritoneal tuberculosis. This report highlights the limitations of relying on immunological tests alone, as IGRAs cannot distinguish latent from active tuberculosis. The subsequent diagnostic delay underscores the importance of integrating clinical, radiological, microbiological, and histopathological findings, with early tissue diagnosis and prompt reassessment when the clinical course is inconsistent with the expected response to empirical therapy, to facilitate timely oncologic management. Early tissue diagnosis should be strongly considered in patients with atypical intra-abdominal masses or in those who fail to respond to empiric therapy. Histopathological and immunohistochemical evaluation remain essential for establishing the diagnosis and guiding appropriate oncologic management. Increased awareness of this rare presentation and a multidisciplinary diagnostic approach may facilitate earlier recognition, reduce avoidable diagnostic delay, and improve clinical decision-making in future cases.
